# Population mobility, deprivation and self-reported limiting long-term illness in small areas across Scotland

**DOI:** 10.1016/j.healthplace.2008.01.009

**Published:** 2009-03

**Authors:** Denise Brown, Alastair H. Leyland

**Affiliations:** MRC Social and Public Health Sciences Unit, 4 Lilybank Gardens, Glasgow G11 8RZ, Scotland

**Keywords:** Mobility, Census output areas, Urban–rural classification, Deprivation, Limiting long-term illness

## Abstract

This study investigates population mobility and its relationship with area level deprivation and health. Based on UK movement in the year preceding the 2001 census, small areas in Scotland were classified as being one of the following population types; decreasing, increasing or stable (with high or low turnover). In the most deprived areas, illness rates for those under 65 were significantly lower in stable populations with low turnover than in other areas of comparable deprivation. Decreasing populations in deprived areas had significantly highest illness rates overall. Leaving those in poor health behind may lead to artifactual increases in area based health inequalities.

## Introduction

Comparison of area populations over time allows changes in population health status and progress towards targeted reductions in inequalities in health to be assessed. Until the 1990s, UK population growth was mainly due to the number of births exceeding the number of deaths. More recently, however, the largest change in population composition has been due to migration (Office for National Statistics, 2004). Migration is the most difficult component of population change to measure as there is no comprehensive registration of migration in the UK. Estimates therefore have to be based on survey and proxy data. The most reliable data currently available to measure population migration come from the UK census. Carried out decennially, it provides the base for population estimates which are updated each year to produce mid-year estimates. At the 2001 census, attention was drawn to the extent to which population movement between censuses had been undetected (General Registrar Office Scotland, 2006). Underestimation of movement, particularly among young men, had led to unreliable population estimates at both large and small area level within the UK. This results in the measurement of changes in population health over time being problematic, since the denominator data are incorrect.

These problems are exacerbated as the characteristics of those who move are not random. Migrants differ from the general population in terms of a number of factors including stage in life course, age, sex, marital status, tenure and ethnicity (Champion et al., 1998). Migration is also selective by health status; however, the relationship is complex. While young migrants are generally healthier than non-migrants, older people are more likely to migrate if they are ill than if they are well in order to be closer to health care services and family (Bentham, 1988; Findley, 1988; Larson et al., 2004). The distance moved by migrants is also an important factor when examining the relationship between health and migration (Fox et al., 1982). After controlling for age, Boyle et al. (2002) found that migrants moving short distances were more likely to report a limiting long-term illness (LLTI) than both long distance migrants or non-migrants. However, while some types of migration are selective of people in poor health, most individuals who migrate will have above average health. As a result, illness and mortality rates will fall in places that are gaining people (Davey Smith et al., 1998, 2001; Boyle et al., 2001; Regidor et al., 2002; Exeter et al., 2005) and rise in places that are losing people. Alternatively, selective migration could lead to an increase in illness and mortality rates of both the origin and destination if the migrant has better health than average in the place they are leaving but worse health than the place they are joining.

If possible, people leave unfavourable physical and social environments to move to more attractive places (Molarius and Janson, 2000). This has resulted, in recent decades, in populations increasing in affluent areas and populations decreasing in deprived areas (Connolly et al., 2007). Previous studies have examined the health status of areas in relation to population mobility and deprivation. O’Reilly (1994), in response to a paper by Phillimore et al. (1994), found that, in all district councils in Northern Ireland, standardised death rates reduced between 1980–1982 and 1990–1992 with the more deprived areas showing the least improvement. The percentage reduction was significantly related to population change due to migration between the 1981 and 1991 censuses. In addition to mortality, Norman et al. (2005), using data from the ONS longitudinal study, looked at self-reported LLTI in relation to mobility and deprivation. They found that over the 20-year period between 1971 and 1991, inequalities in health between the most and least deprived areas had increased. This gradient was particularly steep for LLTI. It was suggested that mobility accounted for most of this change: had the place of residence and deprivation circumstances of individuals not changed over this time period then the observed inequalities would have been less steep. While some have observed geographical inequalities in health to be entirely accounted for by population redistribution (Brimblecombe et al., 1999), others have been unable to confirm that redistribution is the cause of widening health gaps (Boyle et al., 2004b). O’Reilly et al. (2001) point out, however, that given its possible contribution to the changing patterns of health, population mobility should be considered in all ecological studies that aim to make comparisons of area populations over time.

This paper investigates small area population mobility in Scotland, in the year prior to the 2001 census. Studies that have looked at population mobility have tended to do so between censuses and at fairly large geographical area levels. However, in order for population mobility to be taken into account in future analyses the extent to which small areas may change over a relatively short time period needs to be considered. Three general aims are considered:

1. Using an urban–rural classification we investigate whether those populations that changed were most likely to be found in urban or rural Scotland.
2. We investigate whether population mobility in Scotland over this 1-year time period was related to area deprivation.
3. We examine how illness rates, for those under 65 and those aged at least 65 years old, vary according to the extent of population mobility and area deprivation.

## Data and methods

### Population

We use 2001 Scottish census data to assess population mobility in the 42,604 output areas (OAs) of Scotland. The average population size of an OA, the smallest area of census geography, in 2001 was 119 (min: 50, max: 2357). The few larger OAs were due mainly to populations in communal establishments, e.g. prisons, large hospitals and hotels. In total, 1.7% (86,006) of Scotland's population at the 2001 census were communal establishment residents. In order to reduce the influence of large establishments in our analysis, only those persons in private households are included in the population denominator. The average population size of an OA including household residents only was 117 (min: 29, max: 529).

### Mobility

At the 2001, Scottish census respondents were asked ‘What was your usual address one year ago?’ Just under 12% of people resident in Scotland had moved in the previous year, the vast majority from another Scottish address (Fleming, 2005). Multiple moves within the year were not accounted for. Of those moves, 94% were into private household residences. Based on responses given, we are able to examine inflows and outflows for private household residents at an OA level. We restrict this analysis to movement within the UK only since those who left the UK in the year before the census would not have completed a UK census form. Based on UK movement, OAs were classified as one of the following: as a decreasing population (5%+net decrease), as an increasing population (5%+net increase), or as a stable population (<5% total change). Median net population change at the OA level in Scotland in the year prior to the census was 4%. Populations categorised as decreasing or increasing at the 5% level therefore have experienced slightly higher than average net population change. Net population change was also measured at the 10% level as a form of sensitivity analysis and in order to make comparisons between those populations that changed somewhat and those that changed the most. Many of the OAs will be classified as stable, particularly when net population change is measured at the 10% level, however, the term ‘stable’ may be slightly misleading. While some populations remain fairly constant in size, the structure of that population may change. Turnover measures moves in and out of an area in relation to the size of the population and indicates that although the size of the population is not necessarily changing the individuals who make up that population are changing. Although the focus here is on net levels of population change, for stable areas only we make the distinction between areas with high and low turnover. This enables us to compare areas with high levels of net change to areas that remained truly stable (i.e. stable in terms of both size and structure). Hence this gives us four population mobility categories in total. Median turnover at the OA level in Scotland in the year before the census was 16%. We therefore define OAs as being stable populations with high turnover if net population change is less than 5% (or 10%) but turnover is greater than 16%.

### Urban–rural classification

The six-fold Scottish Executive Urban–Rural (SEUR) Classification 2003–2004 (Scottish Executive, 2004b) is used to identify where in Scotland OAs lie. Two main criteria have been used to produce the classification: settlement size as defined by the General Register Office for Scotland (GROS) and accessibility based on drive time analysis. Settlements are classified into large urban areas (settlements of over 125,000 people), other urban areas (settlements of between 10,000 and 125,000 people), small towns (settlements of between 3000 and 10,000 people) and rural areas (settlements of less than 3000 people). Drive times are then estimated to distinguish between accessible and remote small towns and rural areas. A small town or rural area is defined as remote if there is a drive time of over 30 min to a settlement of 10,000 people or more.

### Area level deprivation

Area level deprivation was measured using the Scottish Index of Multiple Deprivation (SIMD) 2004 (Scottish Executive, 2004a). The SIMD 2004 is based on the small area data zone geography, designed to identify pockets of deprivation and is based on census data and other data sources from around 2001. There are 6505 data zones in Scotland (average population 778 people (min: 476, max: 2813)) ranked from most deprived (1) to least deprived (6505). Data zones are aggregations of OAs, with seven OAs on average per data zone, and nest within Scotland's 32 council areas. The overall SIMD rankings are made up of six individual domains: current income; employment; health; education, skills and training; housing; and geographical access and telecommunications. Current income and employment have the largest weightings in the overall SIMD score. Individual domains are also ranked from most deprived (1) to least deprived (6505). The health domain of the SIMD includes an indicator of the comparative illness factor (CIF) based on reporting of general health and LLTI in the 2001 census. As LLTI is our main health measure we prefer not to include this component in the construction of an area deprivation index. Hence we use the current income domain as our measure of area level deprivation instead of the overall rankings.

### Health measure

Self-reported limiting long-term illness (LLTI) was also census derived. Respondents were asked ‘Do you have any long-term illness, health problem or disability which limits your daily activities or the work you can do? Include problems which are due to old age’. The percentage of all private household residents who suffered a self-reported LLTI was 19.7% (compared to 57.8% of all communal establishment residents). Standardised illness rates, for males and females within private households only, are calculated for those aged less than 65 and those aged 65 years and over.

## Results

### Population mobility in Scotland

Overall, Scotland experienced a net loss of around 0.01% of its population to the rest of the UK (Table 1). At small area level, 37% of OAs in Scotland experienced a net decrease or increase in population size of at least 5% due to within UK mobility. The remaining OAs remained stable; that is, they experienced less than 5% change in total population size overall (24% remained stable with high turnover and 39% remained stable with low turnover). For population mobility assessed at the 10% level most OAs remained stable with high (39%) or low (49%) turnover. Only 12% of all OAs had decreased or increased by at least 10% in the year before the census.

### Population mobility in urban and rural areas

In 2001, 68.9% of all OAs lay in urban areas, 13% in small towns and the remaining 18.1% in rural areas (Table 1). OAs ranged from an average size of 113 in large urban areas and remote small towns to 122 in accessible rural areas. The rate of self-reported LLTI was highest in large urban areas and lowest in accessible rural areas (21.1% and 16.8%, respectively, compared to 19.7% in all of Scotland). Urban areas saw a net loss in population in the year prior to the census while small towns and rural areas saw net gains. For population mobility assessed at the 5% level, accessible small towns had the largest proportion of stable populations with low turnover; 43.4% compared to 39% in all of Scotland. Large urban areas contained the highest proportion of increasing areas (20.3% compared to 18.9% in all of Scotland), while remote rural areas contained the highest proportion of decreasing areas (19.3% compared to 17.7% in all of Scotland).

For population mobility assessed at the 10% level, this picture changes slightly. Accessible small towns still contain the largest proportion of stable populations with low turnover (54.4% compared to 49.3% in all of Scotland); however, large urban areas now contain both the highest proportion of increasing areas (8.3% compared to 7.3% in all of Scotland) and the highest proportion of decreasing areas (5.8% compared to 4.6% in all of Scotland).

### Population mobility and area level deprivation

We can also examine the distribution of OAs by quintile of deprivation (Table 1). The average size of an OA ranged from 110 in the most deprived quintile to 129 in the least deprived quintile. Residents in the most deprived quintile reported the highest rate of LLTI and those in the least deprived quintile the lowest rate (27.6% and 12%, respectively, compared to 19.7% in all of Scotland). The most deprived quintile saw the largest net change in population (net loss of 0.54% compared to a net loss of 0.01% in Scotland). When population mobility was assessed at the 5% level, the most deprived quintile contained the largest proportion of decreasing populations (21.5% compared to 17.7% in all of Scotland) and the least deprived quintile contained the largest proportion of stable populations with low turnover (45.7% compared to 39% in all of Scotland).

For population mobility assessed at the 10% level, the most deprived quintile still contained the largest proportion of decreasing populations (7.4% compared to 4.6% in all of Scotland) and the least deprived quintile still contained the largest proportion of stable populations with low turnover (55.1% compared to 49.3% in all of Scotland).

### Population mobility, deprivation and health status in the under 65s

**Fig. 1:**
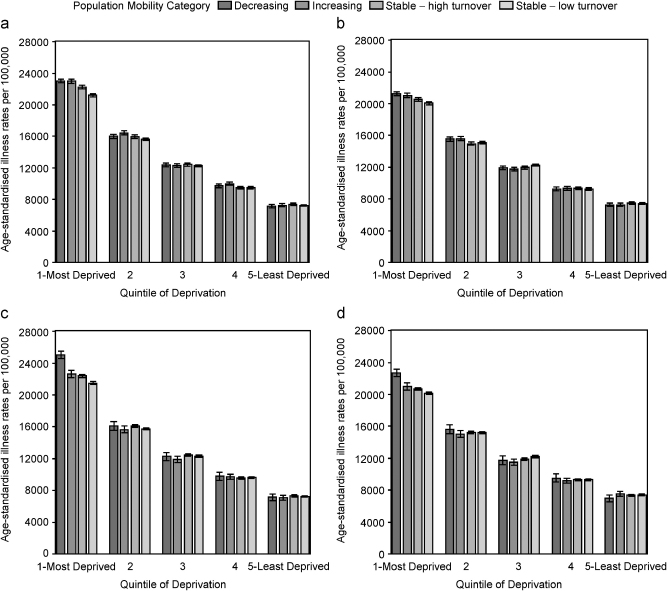
Age standardised illness rates per 100,000 population for males (left-hand side) and females (right-hand side) aged less than 65 years old. The top row corresponds to population change assessed at the 5% level and the bottom row to population change assessed at the 10% level males.

Directly age standardised illness rates, for the resident population as at 2001, were calculated separately, by sex, for those aged under 65 and aged 65 years and older, within each quintile of deprivation by population mobility category. Rates were standardised using the European standard population. For both males and females under 65 (Fig. 1), there was a clear steep illness gradient across the deprivation quintiles. Within deprivation quintiles, for population mobility assessed at the 5% level, there were little, if no, significant differences in illness rates by population mobility category, for males (Fig. 1a) and females (Fig. 1b), in all but the most deprived quintile. For males in the most deprived quintile, illness rates were significantly higher in populations that increased or decreased by at least 5% compared to stable populations. Illness rates were lowest in stable populations with low turnover. Excess illness for those in decreasing populations compared to stable populations with low turnover was 9%. For females in the most deprived quintile, a similar picture emerged. Illness rates were significantly lower in stable populations with low turnover when compared to areas of comparable deprivation. Excess illness for those in decreasing populations compared to stable populations with low turnover was 6%.

When population mobility was assessed at the 10% level, the illness gradient across deprivation quintiles was found to be slightly steeper than for population mobility assessed at the 5% level, for both males (Fig. 1c) and females (Fig. 1d). Comparing population mobility categories within quintiles, there were little or no significant differences in illness rates across all but the most deprived quintile of deprivation. Illness rates in the most deprived quintile were significantly higher for males and females in populations that decreased by at least 10% (illness rate of 25,028 per 100,000 and 22,705 per 100,000, respectively). Excess illness for those in populations that decreased by 10% compared to stable populations with low turnover was 16% for males and 13% for females.

### Population mobility, deprivation and health status in the 65s and over

**Fig. 2:**
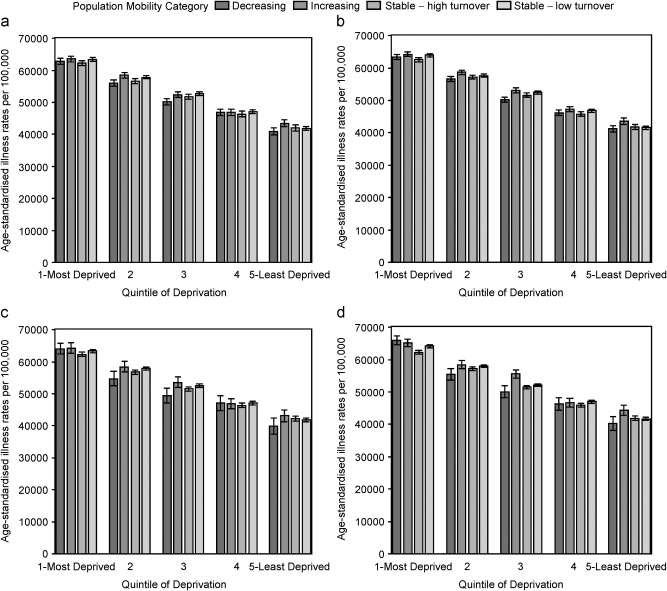
Age standardised illness rates per 100,000 population for males (left-hand side) and females (right-hand side) aged 65 years or older. The top row corresponds to population change assessed at the 5% level and the bottom row to population change assessed at the 10% level.

There was an illness gradient across deprivation quintiles for those aged 65 and over (Fig. 2), although it was less steep than for the under 65s. For population mobility assessed at the 5% level, there was little difference in illness rates when comparing population mobility categories within deprivation quintiles for both males (Fig. 2a) and females (Fig. 2b).

When population mobility was assessed at the 10% level, there was some evidence of differences in illness rates when comparing population mobility categories within deprivation quintiles for males (Fig. 2c) and females (Fig. 2d). Illness rates in the most deprived quintile were lower for males, and significantly lower for females, living in stable populations with high turnover. In the least deprived quintile, illness rates were higher for males and significantly higher for females living in increasing populations. Additionally, in the middle quintile of deprivation, the illness rate for females in increasing populations was significantly higher than in other areas of comparable deprivation. This rate was higher than in decreasing populations in more deprived areas (illness rate of 55,586 per 100,000 in increasing populations in the middle quintile of deprivation compared to illness rate of 55,463 per 100,000 for decreasing populations in deprivation quintile 2), although this difference was not significant.

## Discussion

We examined the whereabouts of different OAs in Scotland, as classified by their population mobility categories, using an urban–rural classification. Despite an expectation that most population mobility would be at the urban area level, we observe little evidence that this is the case. Large urban areas and remote areas (remote small towns and remote rural areas) were found to have the fewest stable, low turnover, populations. OAs in large urban areas were more likely to experience an increase of at least 10% in population size while those in remote areas were most likely to remain stable in size but experience high levels of population turnover.

OAs in the least deprived quintile contain the largest proportion of stable, low turnover, populations while OAs in the most deprived quintile contain the largest proportion of decreasing populations. This observation, for the most deprived quintile, is particularly evident in those OAs that decreased by at least 10%. This confirms previous findings that people are moving away from deprived areas (O’Reilly and Stevenson, 2003), although here we find no evidence of an influx to any specific group of areas which were less deprived.

The majority of OAs experienced less than 5% change in population size due to within UK mobility. Given that most OAs were stable, it was important to differentiate between those where the population size and structure remained the same (stable with low turnover) and those where the population size had remained the same but the structure had changed (stable with high turnover). High and low levels of population turnover are not restricted, however, to stable populations, as areas that experience high levels of net population change may also experience lower than average levels of population turnover. Bontje and Latten (2005), in a study looking at population change in the four largest Dutch cities, found that while the population size in those areas had stabilised in recent years, the population structure had changed, particularly in terms of ethnicity, household type and socio-economic status. This has important implications for the population left behind. Boyle et al. (2004a) showed that as the relative deprivation of the areas that people lived in changed, so too did their mortality and health status. We found evidence that illness rates in the most deprived quintile of deprivation differed, for males and females aged less than 65, in terms of within UK population mobility. Regardless of whether change was assessed at the 5% or 10% level, illness rates for both sexes were significantly lower in stable populations with low turnover when compared to other areas of comparable deprivation. Lower illness rates may reflect the increased social integration and support of stable, low turnover, populations (Sampson, 1988). Areas of low residential mobility have been shown to be independently associated with health benefits such as lower rates of major depression and schizophrenia (Silver et al., 2002) although the association between stable, low turnover, populations and LLTI seen here for under 65s exists only in most deprived areas. Illness rates were highest overall in deprived areas that had decreased by at least 10%, perhaps suggesting that those who moved from decreasing areas may have been healthier than those left behind. This may also help to explain why, in deprived areas, increasing populations and stable population with high turnover had significantly higher illness rates than stable populations with low turnover. If those moving out of decreasing areas have higher illness rates than the areas they join (increasing or high turnover populations) then this would lead to increased illness rates of those areas compared to stable, low turnover, populations.

Differences in the reporting of LLTI by population mobility category were less evident for those aged 65 and over. In the most deprived quintile, illness rates for females were significantly lower in stable populations with high turnover, while in the middle and least deprived quintiles of deprivation illness rates were significantly higher in populations that increased by at least 10%. Similar patterns were found for men; however, differences in illness rates across population mobility categories were non-significant. The explanations for these findings are unclear. Although we have excluded communal establishments from our analysis, perhaps those populations in less deprived areas that increased by at least 10% include areas to which people retire or move in ill health to be closer to family. An influx of unhealthy older people to those areas could result in increased illness rates.

This study has some limitations. The use of LLTI relies on individuals’ perceptions of their own health status and despite being shown to correlate well with all cause mortality (Charlton et al., 1994) geographical differences in self perceived poor health exists (Barnett et al., 2001). It is unclear how long individuals had been suffering from poor health prior to it being reported at the census in 2001 and the environment that caused poor health may have been different to the area lived in by the individual at the time of reporting it. However, given that the illness is defined as being long-term, the onset of illness is unlikely to be related to migration in the previous year. In 2001, the census, for the first time, asked respondents about their general health; ‘Over the last twelve months would you say your health has on the whole been: Good, Fairly good, Not good?’ Although both poor general health (‘not good’) and LLTI were considered as health measures, only the results for LLTI were presented here as similar conclusions were reached for the analysis of general health. Correlation between these two census outcomes has been shown to be high (O’Reilly et al., 2005).

The conclusions only reflect population mobility in Scotland over a 1-year time period. However, looking at change over a 1-year period gives a good indication of the pace of change in Scotland around this time. It is also less likely that multiple moves were made by individuals over this period. Observed changes over a longer time frame may appear more subtle than they actually are; periods of population increase followed by periods of population decrease may result in a population being classified as stable. The findings from this study could be misleading if one off events took place that year, for example major regeneration activities. However, one strength of this study is that the entire population of Scotland is examined. While some OAs may have experienced more population mobility than usual it is unlikely that all OAs in any one population mobility category did.

This paper explores the complex nature of population mobility, illustrating how illness rates may differ according not only to the level of deprivation and the age of the population but also to the levels of population mobility experienced. Population mobility leads to problems with comparisons of areas over time as we are unable to compare like with like. Differences are most marked at the extremes of the deprivation distribution where population shifts may be resulting in increasing illness rates among some of the most deprived areas. This process of residualisation, whereby those in poor health are being left behind, may lead to artifactual increases in area based health inequalities.

## Figures and Tables

**Table 1:** Percentage of OAs in each population mobility category by urban-rural classification and quintile of deprivation

	No. of OAs	Average OA size	LLTI (%)	Net change (%)	5% population mobility				10% population mobility			
					Decreasing (%)	Increasing (%)	Stable-high turnover (%)	Stable-low turnover (%)	Decreasing (%)	Increasing (%)	Stable-high turnover (%)	Stable-low turnover (%)
Urban–rural classification												
Large urban areas	17,167	113	21.1	−0.29	18.6	20.3	24.6	36.5	5.8	8.3	39.9	46.0
Other urban areas	12,189	119	19.7	−0.04	17.3	17.0	24.3	41.4	3.9	6.2	37.9	52.0
Accessible small towns	4297	121	18.8	0.16	15.9	16.9	23.8	43.4	3.0	5.8	36.8	54.4
Remote small towns	1247	113	19.5	0.39	16.6	19.8	28.1	35.5	3.6	7.2	43.9	45.3
Accessible rural	5251	122	16.8	0.62	16.3	19.4	23.5	40.8	3.7	7.9	36.6	51.8
Remote rural	2453	115	17.9	0.06	19.3	20.1	24.9	35.7	4.7	7.4	40.3	47.6
Quintile of deprivation												
Most deprived	9271	110	27.6	−0.54	21.5	19.8	25.7	33.0	7.4	7.2	42.3	43.1
Quintile 2	8823	113	23.2	0.19	16.9	20.1	24.2	38.7	4.2	7.0	38.8	49.9
Quintile 3	8547	115	19.6	0.05	17.1	18.8	24.2	39.9	4.0	6.9	38.8	50.3
Quintile 4	8261	119	15.6	0.35	16.7	19.8	24.5	39.0	3.7	8.4	38.7	49.2
Least deprived	7702	129	12.0	−0.09	15.5	15.7	23.1	45.7	3.2	7.1	34.5	55.1
Scotland	42,604	117	19.7	−0.01	17.7	18.9	24.4	39.0	4.6	7.3	38.8	49.3

Also shown is the total number and average size of OAs within each urban-rural area and quintile of deprivation together with the percentage of respondents reporting an LLTI and the total percentage net population change in that area. Scotland as a whole is shown for comparison.
